# Hand hygiene compliance and its drivers in long-term care facilities; observations and a survey

**DOI:** 10.1186/s13756-022-01088-w

**Published:** 2022-03-18

**Authors:** Anja Haenen, Sabine de Greeff, Andreas Voss, Janine Liefers, Marlies Hulscher, Anita Huis

**Affiliations:** 1 Scientific Center for Quality of Healthcare, Radboud Institute for Health Sciences, Radboud University Medical Centre, P.O. Box 9101, 6500 HB Nijmegen, The Netherlands; 2 Department Antimcrobial Resistance and Healthcare related Infections of the unit Epidemiology and Surveillance of the Centre for Infectious Disease Control of the National Institute, P.O. Box 1, 3720 BA Bilthoven, The Netherlands; 3 Department of Medical Microbiology, Canisius-Wilhelmina Hospital, P.O. Box 9015, 6500 GS Nijmegen, The Netherlands

**Keywords:** Hand hygiene, Long-term care facilities, Nursing staff, Nursing professional, Questionnaire

## Abstract

**Background:**

Hand hygiene is an important measure to prevent healthcare-associated infections in long-term care facilities.

**Objectives:**

To evaluate compliance with hand hygiene recommendations by different nursing professionals in long-term care facilities and to investigate determinants potentially influencing hand hygiene and whether these differed between the different cadres of staff.

**Methods:**

We conducted two sub-studies: we measured hand hygiene compliance of 496 professionals in 14 long-term care facilities (23 wards) through direct observation using World Health Organisation’s ‘five moments of hand hygiene’ observation tool. In addition, we performed a survey to examine determinants that may influence hand hygiene and to determine differences between different cadres of staff. We used a principal component analysis approach with varimax rotation to explore the underlying factor structure of the determinants.

**Results:**

We found an overall mean hand hygiene compliance of 17%. There was considerable variation between wards (5–38%) and between specific World Health Organization hand hygiene moments. In addition, hand hygiene compliance varied widely within and between different cadres of staff. The determinant analysis was conducted on 177 questionnaires. For all nursing professionals, we found multiple determinants in four domains: ‘social context and leadership’, ‘resources’, ‘individual healthcare professional factors’ and ‘risk perception’. In two domains, several barriers were perceived differently by nursing assistants and nurses. In the domain ‘social context and leadership’, this included (1) how the manager addresses barriers to enable hand hygiene as recommended and (2) how the manager pays attention to correct adherence to the hand hygiene guidelines. In the ‘risk perception’ domain, this included a resident's risk of acquiring an infection as a result of the nursing professional’s failure to comply with the hand hygiene guidelines.

**Conclusion:**

Hand hygiene compliance was low and influenced by multiple factors, several of which varied among different cadres of staff. When designing interventions to improve hand hygiene performance in long-term care facilities, strategies should take into account these determinants and how they vary between different cadres of staff. We recommend exploring hand hygiene determinants at ward level and among different cadres of staff, for example by using our exploratory questionnaire.

**Trial registration:**

Registration number 50-53000-98-113, ‘Compliance with hand hygiene in nursing homes: go for a sustainable effect’ on ClinicalTrials.gov. Date of registration 28-6-2016.

**Supplementary Information:**

The online version contains supplementary material available at 10.1186/s13756-022-01088-w.

## Background

Adherence to hand hygiene recommendations is important to prevent healthcare-associated infections in all settings but especially in care institutions for the elderly such as long-term care facilities [1]. In long-term care facilities, microorganisms can easily be transmitted because most residents use shared facilities, live in close proximity, and have close relationships with other residents and nursing staff. Several studies of long-term care facilities have demonstrated the effectiveness of hand hygiene in removing pathogens from the hands of healthcare workers and reducing infection rates [2–6]. There are only a few studies quantifying hand hygiene compliance in long-term care facilities, but generally they show that compliance in daily practice is low, ranging from 11 to 26% [7–11]. Moreover, Mills et al. (2019) concluded that hand hygiene compliance was influenced by job title [12]. In their study, certified nurse assistants showed lower hand hygiene compliance than registered nurses.

A wide range of nursing-professional-related barriers to hand hygiene have been identified in long-term care facilities, such as beliefs about negative consequences, lack of knowledge and lack of hand hygiene training [13–17]. Resident-related factors such as unpredictable resident behaviour and unwillingness to receive care may also influence hand hygiene [13]. Healthcare professionals in long-term care facilities constantly pursue a balance between working hygienically, responding adequately to acute care needs and maintaining a homelike environment for their residents. Moreover, (1) professional communication and interactions; (2) the availability, accessibility, and content of guidelines and (3) the availability of incentives and resources are important factors helping or hindering hand hygiene [13]. It is unclear, however, whether potential determinants (barriers and facilitators) of hand hygiene differ for nursing professionals with different job titles and tasks.

In this study, we investigated hand hygiene compliance in Dutch long-term care facilities for specific nurse professional groups (i.e., nurses who provide high-complexity care, nurse assistants who provide low-complexity care and support, care assistants who help with bathing, dressing and grooming and housekeeping assistants who perform household chores). In addition, we explored determinants potentially influencing hand hygiene and whether these differed between the different cadres of staff.

## Methods

### Study design

This multicentre study, performed in 2017, encompassed two sub-studies: (1) an observational study on the performance of hand hygiene and (2) a survey on determinants potentially influencing this performance. The research was part of the study ‘Compliance with hand hygiene in nursing homes: go for sustainable effect’ which aimed to improve hand hygiene in long-term care facilities (The Netherlands Organisation for Health Research and Development 2014, study ID 522002009). The current study reports the baseline hand hygiene compliance and the determinants (barriers and facilitators) influencing hand hygiene compliance among different cadres of staff (nurses, nurse assistants, care assistants, and housekeeping assistants) in long-term care facilities for the elderly.

### Setting and participants

A total of 23 wards with 496 nursing professionals (a mean of 20 nursing professionals per ward with a range of 7–50) in 14 long-term care facilities participated in the study from March 2017 to June 2017. They were recruited from the Dutch national sentinel surveillance network for infectious diseases in long-term care facilities. When interested, the long-term care facilities decided which department(s) participated in the study. Residents in these long-term care facilities have their own rooms, but often share a bathroom and use a communal living room. The 23 wards represented psycho-geriatric (9), somatic (8) and rehabilitation care (6). The study population consisted of nurses, nurse assistants, care assistants, housekeeping assistants, and the trainees of the different professions, all with different educational levels and general tasks (Table 1),

**Table 1:** Professional status, educational level and general task description by profession

Profession	Educational level	General task description
Nurse	Level 4 and 5	(High complexity) nursing and care
Nurse assistants	Level 3	Low complexity nursing care and support
Care assistants	Level 2	Bathing, dressing and grooming
Housekeeping assistants	Level 1	Household chores

According to Dutch Qualification Framework (2019)

### Variables and data collection

#### Sub-study 1: observational study on hand hygiene compliance

Per ward, 12–15 nursing professionals were observed for 20 min according to the World Health Organization’s hand hygiene monitoring tool including and at least four opportunities for hand hygiene per nursing professional. If necessary, this observation period was extended. Direct, unobtrusive observations were used to measure hand hygiene performed by nursing professionals at the participating wards. To mask the true subject of the observation, the observers stated they were observing patient safety topics. The observations were conducted during morning care (i.e. a moment of the day with a high density of care with activities such as bathing and dressing and wound care and the like) to efficiently collect a large number of opportunities and obtain a clear picture of all hand hygiene moments. Each nursing professional was observed only once in this study. The observed moments included hand hygiene (1) before touching a resident*,* (2) before a clean or aseptic procedure, (3) after body-fluid-exposure risk, (4) after touching a resident and (5) after touching residents’ surroundings [18]. Hand hygiene was operationalized as ‘hand washing with either plain soap and water’ or ‘hand disinfection through the use of an alcohol-based hand rub solution’.

Ten students from three universities of applied sciences (nurses and students of infection and contamination control) were trained as observers. All students participated in a two-day training course to understand the ‘five moments for hand hygiene’ and to apply the World Health Organization’s observation method. Following this training, the observers performed an observation round in a non-participating long-term care facility with an experienced observer to ensure concordance between the observers.

Data were collected using the Observe app of HARTMANN, a monitoring tool that includes the World Health Organization’s ‘five moments of hand hygiene’ for the long-term care facility setting [18]. The observer registered the opportunity for hand hygiene and whether hand hygiene was performed by hand disinfection or hand-washing or was not performed in accordance with the World Health Organization’s ‘five moments for hand hygiene’.

#### Sub-study 2: survey of determinants potentially influencing hand hygiene performance

We developed a questionnaire to measure determinants of hand hygiene performance based on information from focus groups [13]; in these focus groups, we explored potential determinants guided by a generic checklist for identifying determinants of practice [19]. The questionnaire was supplemented with specific hand hygiene items from a questionnaire used in a previous hand hygiene study (‘Helping Hands’) in Dutch hospitals [20]. The questionnaire encompassed 52 statements in six domains: ‘Guideline factors’ (5 items), ‘Individual healthcare professional factors’ (21 items), ‘Patient factors’ (2 items), ‘Professional interactions’ (5 items), ‘Incentives and resources’ (8 items) and ‘Capacity for organisational change’ (11 items). The 52 statements concerned facilitators and barriers potentially influencing hand hygiene compliance. Statements were appraised on a four-point Likert scale (4 = totally agree, 3 = agree, 2 = disagree and 1 = totally disagree). The higher the percentage agreement with a statement referring to a facilitator, the more professionals felt facilitated in complying with hand hygiene. In contrast, the higher the percentage of agreement with a statement referring to a barrier, the more professionals felt hindered in complying with hand hygiene (Additional file 1: Questionnaire to explore the determinants of hand hygiene in Long-term care facilities. docx). The questionnaire was digitalized with LimeSurvey, ensuring anonymized responses.

Each nursing professional, from the 23 wards that participated in the hand hygiene observations, received a link to the questionnaire via his or her e-mail address as provided by the ward’s study contact person. We sent a reminder to all nursing professionals after two weeks. To further increase responses, a small gift was promised to the ward that returned the highest number of questionnaires.

### Data analysis

We analysed the main outcomes, ‘hand hygiene compliance’ and ‘determinants that potentially influence hand hygiene performance’, separately.

#### Sub-study 1: observational study on hand hygiene compliance

Multivariate analysis was performed to study the association between profession and adherence to hand hygiene. We used a linear mixed model with random effects for nursing professional and ward and a fixed effect for profession. However, the model fit was insufficient. There was inconsistency: a nursing professional group performed very well in one ward while the same nursing professional group performed less well in another ward. Therefore, no statistical test was conducted for the association between profession and adherence to hand hygiene. Instead, we used descriptive statistics. The hand hygiene compliance rate was calculated by dividing the number of observed hand hygiene opportunities where hand hygiene was performed by the total number of observed hand hygiene opportunities. We calculated the overall compliance of all long-term care facilities, compliance per ward, compliance per World Health Organization moment per ward and compliance per nursing professional group per ward (mean and standard deviation).

#### Sub-study 2: survey on determinants potentially influencing hand hygiene performance

The questionnaire item scores were recoded as dichotomous scores: ‘agree’ (answer categories ‘totally agree’ and ‘agree’) or ‘disagree’ (answer categories ‘totally disagree’ and ‘disagree’).

A principal component analysis approach with varimax rotation was performed to explore the underlying factor structure of the questionnaire, accepting a factor loading of > 0.4 as sufficient. Statements with little variation (i.e. where > 85% of the respondents agreed or disagreed, n = 23) had no correlation with the other variables and therefore were not included in the factor analysis. Four factors (domains) were identified: ‘Determinants regarding social context and leadership’ (10 questions, α = 0.8), ‘Determinants regarding resources’ (four questions, α = 0.7), ‘Determinants regarding individual healthcare professional factors’ (four questions, α = 0.5) and ‘Determinants regarding risk perception’ (two questions, α = 0.3, Table 2). Together, the four factors (domains) explained 48.5% of the variance.

**Table 2:** Hand hygiene opportunities and compliance per World Health Organization hand hygiene moment and by profession

	Opportunities N (n wards)	Hand hygiene compliance (range between wards %)	Standard deviation
*World Health Organization hand hygiene moments*			
Before touching a resident	519 (23)	18% (0–54)	0.14
Before a clean or aseptic procedure	43 (17)	9% (*)	0.18
After body-fluid-exposure risk	663 (23)	10% (0–28)	0.1
After touching a resident	576 (23)	25% (4–70)	0.42
After touching residents’ surroundings	128 (22)	11% (*)	0.16
*Hand hygiene opportunities and compliance per profession*			
Nurses (n = 59)	365 (20)	24% (0–58)	0.17
Nurse assistants (n = 124)	797 (23)	18% (0–50)	0.13
Trainees (n = 51)	329 (18)	17% (0–100)	0.23

*Not shown due to low numbers of opportunities per ward

We used a two-sample t-test to determine if there was a statistically significant difference between the means per factor (i.e. the domain mean scores) of nurses and nurse assistants, incorporating only the questionnaires from these professional groups. Subsequently, we calculated the percentage of nurses and nurse assistants who agreed with each statement (overall and per professional group) and the difference in percentages between the two professions.

Analysis of the data was undertaken using the Statistical Package for the Social Sciences (version 25.0).

### Trial registration

Registration number 50-53000-98-113, ‘Compliance with hand hygiene in nursing homes: go for a sustainable effect’ on ClinicalTrials.gov. Date of registration 28-6-2016.

## Results

### Sub-study 1: observational study on hand hygiene compliance

On average, we observed 12 nursing professionals on each ward (range 8–17) for at least four hand hygiene opportunities per person. We observed 1691 opportunities on 23 wards of 14 long-term care facilities: 519 opportunities before touching a resident, 43 before a clean or aseptic procedure, 663 after body-fluid-exposure risk, 576 after touching a resident and 128 after touching residents’ surroundings. In practice, multiple opportunities have occurred simultaneously.

**Fig. 1:**
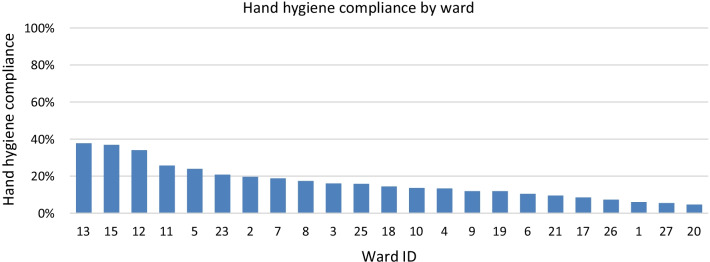
Hand hygiene compliance by ward

The overall mean hand hygiene compliance was 17% (standard deviation = 0.1) and varied widely between wards, ranging from 5 to 38% per ward (Fig. 1).

Hand hygiene compliance was highest ‘after touching a resident’ (25%, standard deviation = 0.42) and lowest ‘before a clean or aseptic procedure’ (9%, standard deviation = 0.18). The mean compliance per World Health Organization moment varied from ward to ward (Table 2).

The 1691 hand hygiene opportunities were observed in 271 healthcare professionals: 124 nurse assistants (23 wards, 797 opportunities), 59 nurses (20 wards, 365 opportunities), 28 housekeeping assistants (13 wards, 162 opportunities), nine care assistants (five wards, 38 opportunities) and 51 trainees (18 wards, 329 opportunities). Due to low numbers of other nursing professions, only data of nurses, nurse assistants and trainees are reported here.

The overall mean hand hygiene compliance varied from 17% for trainees to 24% for nurses (Table 2). Again, large differences were seen between wards.

### Sub-study 2: survey on determinants potentially influencing hand hygiene performance

We received 196 questionnaires (response rate: 40%) from 22 wards in 14 long-term care facilities. For 19/196 questionnaires, the ward identity was not filled in and these questionnaires were not included in the study. For 11/177 questionnaires, less than 80% of the questions had been completed; these questionnaires were not included in the principal component analysis but were included in the further analyses. At least 10% of the nursing professionals of each ward in the observational study (range: 4–15 professionals) completed a questionnaire. The 177 questionnaires (response rate: 36%) were from 92 nurse assistants (from 22 wards of 14 long-term care facilities, range: 1–9 per ward), 56 nurses (from 19 wards of 13 long-term care facilities, range: 1–7 per ward), four care assistants (from four wards of four long-term care facilities), 10 trainees (from six wards of four long-term care facilities, range: 1–3 per ward) and 15 housekeeping assistants (from 10 wards of eight long-term care facilities, range: 1–5 per ward).

Given the low numbers of the other different cadres of staff, we report only the questionnaire results for nurses and nurse assistants. Table 3 describes the level of agreement with the various statements within the four domains (social context and leadership, resources, individual healthcare professional factors and perception) for nurse assistants and nurses combined (overall agreement) and for nurses and nurse assistants separately.

**Table 3:** Results of the determinant questionnaire

Domain	Statements Facilitator: preferably as high an agreement percentage as possible Barrier: preferably as low an agreement percentage as possible	% agree overall (nurse assistants and nurses)	% agree nurse assistants	% agree nurses	Δ% agree (nurse assistants versus nurses)
Social context and leadership	My manager holds team members accountable for hand hygiene performance (Facilitator: Leadership)	64	69	55	13
	Infection prevention is an important issue in my ward (Facilitator: Social context)	74	74	74	0
	In my ward, colleagues support each other in adhering to the hand hygiene guidelines (Facilitator: Social context)	68	71	63	8
	My manager addresses barriers to enable hand hygiene as recommended (Facilitator: Leadership)	69	78	54	24*
	My manager regularly pays attention to adhering to the hand hygiene guidelines (Facilitator: Leadership)	49	56	38	18*
	The residents in my ward think I should always follow the hand hygiene guidelines (Facilitator: Social context)	70	72	66	6
	If I notice a colleague not complying to the hand hygiene guidelines, I will call him or her on it (Facilitator: Social context)	75	76	74	2
	Our team is informed about our hand hygiene performance (Facilitator: Leadership)	40	43	36	7
	In my ward, we regularly pay attention to the correct application of hand hygiene (Facilitator: Leadership)	63	68	55	13
	My colleagues strictly adhere to the hand hygiene guidelines (Facilitator: Social context)	79	84	71	13
Resources	In my ward, the access to the sink is hindered by a variety of materials and objects, which makes it difficult to apply hand hygiene (Barrier)	29	29	30	-1
	It happens frequently that supplies of soap and towels are not replenished (Barrier)	34	33	36	-3
	It happens frequently that supplies of alcohol-based hand rubs (bottles/dispensers) are not replenished (Barrier)	34	33	34	-1
	There is a shortage of staff in my ward (Barrier)	59	57	63	-6
Individual healthcare professional factors	Correct application of hand hygiene hampers a good relationship with a resident (Barrier)	16	18	13	5
	Sometimes, I don't consider or forget to apply hand hygiene (Barrier)	34	32	36	-4
	Applying hand hygiene is harmful for my skin (Barrier)	37	38	36	2
	I will not interrupt a nursing procedure to apply hand hygiene (Barrier)	22	23	20	3
Risk perception	The risk of a resident acquiring an infection as a result of my failure to comply with the hand hygiene guidelines is not very high (Barrier)	40	28	59	31*
	The risk of a resident acquiring an infection in my ward is not very high (Barrier)	67	67	66	− 1

*Statistically significant

#### Social context and leadership

The statements in this first domain (10 items) all concerned facilitators potentially influencing hand hygiene compliance. This means that the higher the percentage of agreement, the more professionals feel facilitated in complying with hand hygiene. About two thirds of all respondents agreed with the statements within this domain with the exception of two statements related to ‘leadership’: 40% agreed with the statement ‘Our team is informed about our hand hygiene performance’ and 49% agreed with the statement ‘My manager regularly pays attention to adhering to the hand hygiene guidelines’.

Although the mean domain score showed no statistically significant difference between the different cadres of staff, nurse assistants were overall more likely than nurses to agree with statements within the ‘social context and leadership’ domain. A statistically significant difference was found for two leadership statements. Seventy-eight percent of the nurse assistants versus 54% of the nurses agreed with the statement ‘My manager addresses barriers to enable hand hygiene as recommended’ (Δ24%, *p* = 0.033). Fifty-six percent of the nurse assistants versus 38% of the nurses agreed with the statement ‘My manager regularly pays attention to correct adherence to the hand hygiene guidelines’ (Δ18%, *p* = 0.003).

#### Resources

The four statements in the second domain, ‘resources’, all concerned barriers potentially influencing hand hygiene compliance. This means that the higher the percentage of agreement, the more professionals felt hindered in complying with hand hygiene. About one third of the respondents experienced problems related to access to sinks and supplies of towels, soap and hand rub. Fifty-nine percent of the nurse assistants and nurses combined experienced staff shortage. For all barriers and for the domain mean score, no relevant differences were seen between the nurse assistants and nurses.

#### Individual healthcare professional factors

The four statements in the domain ‘individual healthcare professional factors’ all concerned barriers. A minority of the respondents (16%) agreed that hand hygiene hampers a good relationship with a resident. About one-fifth of both nurse assistants and nurses agreed that they would not interrupt a nursing procedure to apply hand hygiene. About one third of both nurse assistants and nurses agreed to sometimes disregarding or forgetting to apply hand hygiene or to considering hand hygiene harmful to their skin. We found no statistically significant differences in the agreement scores between the nurses and nurse assistants nor in the domain mean scores.

#### Risk perception

The two statements in the fourth domain, ‘risk perception’, both concerned barriers. Two thirds of the respondents (both nurse assistants and nurses) agreed with the statement, ‘The risk of a resident acquiring an infection in my ward is not very high’. Forty percent felt that the risk of a resident acquiring an infection as a result of their failure to comply with the hand hygiene guidelines was not very high, with nurse assistants being statistically significantly more aware of this risk than nurses (28% versus 59% agreeing with the statement, *p* = 0.000).

## Discussion

This study shows that overall hand hygiene compliance in long-term care facility wards was low, with substantial variation between wards, between World Health Organization hand hygiene moments and between different cadres of staff. In addition, hand hygiene compliance was influenced by several determinants, some of which varied by professional group.

The low overall mean hand hygiene compliance of 17% from our study is in line with findings from previous studies that reported compliance rates varying from 11 to 26% [9–11]. The recently published study by Teesing et al. (2021) in Dutch long-term care facilities showed a similar low average compliance of 13% [7]. Our study also demonstrated considerable differences in overall hand hygiene compliance between the various wards, ranging from 5 to 38%. Although nurses performed slightly better overall (24%) than nurse assistants (17%) and trainees (18%), large variation between wards was shown with, in some wards, nurse assistants outperforming nurses or vice versa. These findings are consistent with those in other studies [9, 12, 17].

Our study showed large differences in compliance for the different World Health Organization hand hygiene moments. Liu et al. (2014) also found better hand hygiene compliance ‘after patient contact’ than ‘before patient contact’ in long-term care facilities [9]. In contrast to Liu’s et al. study, which demonstrated a hand hygiene compliance rate of 33% ‘after body-fluid-exposure risk’, our study showed a compliance rate of only 10%. Several hospital studies reported that hand hygiene is most often performed after tasks perceived as dirty and that personal protection appeared to be more important for compliance than patient safety [21–23]. A possible explanation for the anomalous finding in our study might be the use of gloves. Wearing gloves may lead to an inappropriate sense of safety and thus eliminate the urge for performing hand hygiene. The study of Teesing et al. (2021) also demonstrated that glove use is a main risk factor for noncompliance with hand hygiene in long-term care facilities. Wearing gloves appeared to be a substitute for hand hygiene, resulting in lower compliance rates compared to not wearing gloves [7]. We therefore recommend dedicated glove-use training for nursing staff in long-term care facilities. Table 4 summarizes the recommendations of our study.

**Table 4:** Recommendations for a successful hand hygiene improvement program in long-term care facilities

Recommendations to be included in making a successful program to improve hand hygiene in long-term care facilities;
Take care of dedicated glove-use training for nursing staff in long-term care facilities
Include strategies aimed at enhancing leadership, tailored to the specific needs of the various professional groups in long-term care facilities
Explore hand hygiene determinants in long-term care facilities at ward level and within professional groups

Within four domains, we found several determinants (barriers and facilitators) potentially influencing the performance of hand hygiene. Regarding ‘social context and leadership’, 40% of our respondents felt they were informed about their hand hygiene performance. Ashraf et al. (2010) also found that half of their surveyed healthcare professionals in long-term care facilities did not receive feedback on their hand hygiene performance [16]. However, feedback is a powerful tool in raising awareness and has been demonstrated to be effective in hand hygiene improvement studies in both hospital and long-term care settings [11, 20]. Half of the respondents in our study felt that their manager regularly paid attention to adhering to the hand hygiene guidelines. Leadership was also recognized in the study of Hammerschmidt et al. (2019) as a facilitator; 73% of the nurse assistants and 64% of the nurses considered their manager a role model [14]. It is noteworthy that managers in their study were not aware of their key role in facilitating the hand hygiene process. In our study, nurse assistants were statistically significantly more likely to report that their manager regularly paid attention to adhering to the hand hygiene guidelines and that their manager addressed barriers to enable hand hygiene as recommended. Therefore, we recommend that hand hygiene improvement programs in long-term care facilities include strategies aimed at enhancing leadership, tailored to the specific needs of the various professional groups (Table 4).

About one third of all respondents had trouble accessing sinks and regularly encountered non-refilled materials such as soap, alcohol-based hand rub and towels. Both Hammerschmidt et al. (2019) and Ashraf et al. (2010) reported difficulties in performing hand hygiene due to a lack of alcohol-based hand rub, soap and towels. It goes without saying that supplies should always be available and that clear arrangements must be made for the replenishment of materials. Almost 60% of all respondents in our study reported staff shortage. Understaffing and high workload are frequently mentioned barriers in hand hygiene studies across all healthcare settings [14, 16, 24]. Unfortunately, staff shortage and high workload are difficult problems to address. Therefore, innovative strategies are needed to ensure that hand hygiene is not viewed as an additional burdensome activity but as an integral part of patient care. It may help if more staff members carry a pocket bottle with alcohol-based hand rub. This means they are not reliant on refilled dispensers, and they are able to perform hand hygiene at any time and in any place. In addition to hand hygiene improvement activities are best incorporated into daily practice and existing work meetings.

In summary, the results of our study show that interventions should focus on the presence and availability of resources and materials. This also applies to the availability of sufficient personnel. With the increasing tightness in the labour market, the latter will be more difficult to achieve, and creative solutions will be needed.

With regard to individual nursing professional factors, our results are consistent with previous research [14, 16]. Forgetfulness and fear of skin damage was reported by more than 34% of our respondents. In addition, 22% said they would not interrupt a nursing procedure to apply hand hygiene. Nursing staff in the study of Hammersmidt et al. (2019) also felt that it is not possible to disinfect your hands while taking care of a resident. In this case, nursing staff could be supported by on-the-job training. Sasahara et al. (2021) successfully trained nursing staff specifically on when hand hygiene should be practiced in conjunction with the daily care tasks in long-term care facilities. Nurses then reflected on their own performance and identified for themselves situations in which hand hygiene is often overlooked [11].

Finally, the remaining domain is risk perception. Two thirds of all respondents believed that the risk of a resident acquiring an infection on their ward was not very high. However, nurse assistants (72%) were more aware that residents may acquire infections due to a lack of hand hygiene than nurses (41%). The reason for this difference in perception is not clear. Nevertheless, low risk perception is problematic and is also reported in the study of Hammerschmidt et al. (2019). They assume that low risk perception is partly driven by the belief that a long-term care facility should reflect a home-like environment rather than a health care facility [14]. The current COVID-19 pandemic has proven otherwise; long-term care facilities all over the world have been disproportionately affected by massive outbreaks, resulting in a dramatic number of deaths [25]. It is crucial to increase current risk perception and awareness and to pay continuous attention to these factors in daily practice. It remains to be seen whether the of professionals’ risk perception has changed permanently after COVID-19.

Our study has several limitations. The questionnaire was completed anonymously to increase the response rate, and therefore we could not directly relate the survey results to individuals’ hand hygiene compliance. However, with both the questionnaire respondents and the observed professionals, we knew which nursing professional group they represented. This allowed us to calculate hand hygiene compliance by nursing professional group and to establish differences in perceived determinants across the different cadres of staff.

Our observations were performed unobtrusively, but some members of the nursing staff may have been aware that they were being observed, possibly resulting in improved hand hygiene. This means that the actual hand hygiene compliance could be even lower than observed in this study.

Finally, participation in the survey was voluntary, and it is possible that mainly respondents with a positive attitude and sufficient knowledge of hand hygiene completed the questionnaire. The nurses and nurse assistants who completed the questionnaire represented, however, almost all participating wards of all long-term care facilities. We therefore expect to have obtained a complete picture of the determinants experienced by these professionals.

## Conclusion

In conclusion, overall hand hygiene compliance in long-term care facilities was low and varied widely for the five World Health Organization moments, between wards and between professional groups. In addition, we identified various determinants of performing hand hygiene. Determinants related to leadership and risk perception were perceived statistically significantly differently by different cadres of staff. Hand hygiene improvement programmes in long-term care facilities should include strategies aimed at improving leadership and risk perception. Team members should be supported by on-the-job training and encouraged to reflect on their own hand hygiene practices and to discuss maintenance of hand hygiene focus. In addition, activities to improve hand hygiene should be incorporated into daily practice and existing work meetings. Given potential differences in other countries and settings, we recommend exploring hand hygiene determinants in long-term care facilities at ward level and within professional groups (Table 4). Barriers and facilitators per ward or profession or both can be identified with our exploratory questionnaire, which addresses the domains ‘social context and leadership’, ‘resources’, ‘individual healthcare professional factors’ and ‘risk perception’.

## Supplementary Information

**Additional file 1.** Questionnaire to explore the determinants of hand hygiene in Long-term care facilities.

## Declarations

### Availability of data and materials

The datasets used and analysed during the current study are available from the corresponding author on reasonable request.

## References

[1] WHO. WHO guidelines on hand hygiene in health care: first global patient safety challenge clean care is safer care. 2009. 23805438

[2] Schweon SJES, Kirk J, Rowland DY, Acosta C (2013). Effectiveness of a comprehensive hand hygiene program for reduction of infection rates in a long-term care facility. Am J Infect Control.

[3] Mody LMS, Sun R, Bradley SF, Kauffman CA (2003). Introduction of a waterless alcohol-based hand rub in a long-term-care facility. Infect Control Hosp Epidemiol.

[4] Yeung WKTW, Wong TW (2011). Clustered randomized controlled trial of a hand hygiene intervention involving pocket-sized containers of alcohol-based hand rub for the control of infections in long-term care facilities. Infect Control Hosp Epidemiol.

[5] Fendler EJAY, Hammond BS, Lyons MK, Kelley MB, Vowell NA (2002). The impact of alcohol hand sanitizer use on infection rates in an extended care facility. Am J Infect Control.

[6] Makris ATML, Gaber DJ, Richter A, Rubino JR (2000). Effect of a comprehensive infection control program on the incidence of infections in long-term care facilities. Am J Infect Control.

[7] Teesing GRRJ, Erasmus V, Petrignani M, Koopmans M, Vos MC, Schols MGA, Voeten H (2021). Hand hygiene and glove use in nursing homes before and after an intervention. Infect Control Hosp Epidemiol.

[8] Ho MLSW, Wong LC, Wong TY (2012). Effectiveness of multifaceted hand hygiene interventions in long-term care facilities in Hong Kong: a cluster-randomized controlled trial. Infect Control Hosp Epidemiol.

[9] Liu WILS, Wu SF, Chuang YH (2014). Hand hygiene compliance among the nursing staff in freestanding nursing homes in Taiwan: a preliminary study. Int J Nurs Pract.

[10] Teppei SKK, Akio Y, Ryusuke A, Dai A, Masanori O, Yuji M (2021). Improvement of hand hygiene adherence among staff in long-term care facilities for elderly in Japan. J Infect Chemother.

[11] Sasahara TKK, Yoshimura A, Ryusuke A, Akine D, Ogawa M, Morisawa Y (2021). Improvement of hand hygiene adherence among staff in long-term care facilities for elderly in Japan. J Infect Chemother.

[12] Mills JPZZ, Mantey J, Hatt S, Patel P, Kaye KS, Gibson K, Cassone M, Lansing B, Mody L (2019). The devil is in the details: factors influencing hand hygiene adherence and contamination with antibiotic-resistant organisms among healthcare providers in nursing facilities. Infect Control Hosp Epidemiol.

[13] Lescure D, Haenen A, de Greeff S (2021). Exploring determinants of hand hygiene compliance in LTCFs: a qualitative study using Flottorps’ integrated checklist of determinants of practice. Antimicrob Resist Infect Control.

[14] Hammerschmidt JMT (2019). Nurses’ knowledge, behaviour and compliance concerning hand hygiene in nursing homes: a cross-sectional mixed-methods study. BMC Health Serv Res.

[15] Smith JDCK, MacDonald TK, Fabrigar LR, Saedi A, Chaplin A (2018). Application of the theoretical domains framework to identify factors that influence hand hygiene compliance in long-term care. J Hosp Infect.

[16] Ashraf MSHS, Agarwal N, Ashraf S, El-Kass G, Hussain R (2010). Hand hygiene in long-term care facilities: a multicenter study of knowledge, attitudes, practices and barriers. Infect Control Hosp Epidemiol.

[17] Aiello AEMM, Knapp JK, Mody L (2009). The influence of knowledge, perceptions, and beliefs, on hand hygiene practices in nursing homes. Am J Infect Control.

[18] WHO. Hand hygiene in outpatient and home-based care and long-term care facilities. 2012.

[19] Flottorp SAOA, Krause J, Musila NR, Wensing M, Godycki-Cwirko M (2013). A checklist for identifying determinants of practice: a systematic review and synthesis of frameworks and taxonomies of factors that prevent or enable improvements in healthcare professional practice. Implement Sci.

[20] Huis AHG, van Achterberg T, Grol R, Schoonhoven L, Hulscher M (2013). Explaining the effects of two different strategies for promoting hand hygiene in hospital nurses: a process evaluation alongside a cluster randomised controlled trial. Implement Sci.

[21] Chang NNSRH, Schweizer ML, Jones I, Chrischilles E, Chorazy M, Huskins C, Herwaldt L (2021). Hand hygiene compliance at critical points of care. Clin Infect Dis.

[22] Santana SLFG, Coutinho AP, Medeiros EA (2007). Assessment of healthcare professionals’ adherence to hand hygiene after alcohol-based hand rub introduction at an intensive care unit in Sao Paulo, Brazil. Infect Control Hosp Epidemiol.

[23] Richards MJRP (2007). Surveillance of hospital-acquired infections in Australia—one nation, many states. J Hosp Infect.

[24] Erasmus VDT, Brug H, Richardus JH, Behrendt MD, Vos MC, van Beeck EF (2010). Systematic review of studies on compliance with hand hygiene guidelines in hospital care. Infect Control Hosp Epidemiol.

[25] Thompson DCBM, Beiu C, Popa LG, Mihai MM, Berteanu M, Popescu MN (2020). The impact of COVID-19 pandemic on long-term care facilities worldwide: an overview on international issues. Biomed Res Int..

