# Supplementary material for: Hand hygiene compliance and its drivers in long-term care facilities; observations and a survey
Source: Antimicrob Resist Infect Control. 2022 Mar 18;11:50. doi: 10.1186/s13756-022-01088-w (PMC8931571; doi:10.1186/s13756-022-01088-w)
Supplement: Supplementary file 1 — Additional file 1. Questionnaire to explore the determinants of hand hygiene in Long-term care facilities. [file 13756_2022_1088_MOESM1_ESM.docx]

| **Questionnaire to explore determinants of hand hygiene in LTCFs** |
| --- |
| The risk of a resident acquiring an infection in my ward is not very high |
| Applying hand hygiene is only important during outbreaks |
| Adhering to the hand hygiene guidelines is not a priority for me |
| I will not interrupt a nursing procedure to apply hand hygiene |
| When it comes to hand hygiene, I do whatever my colleagues do |
| I am aware of the number of infections that occur in the residents of our ward |
| The risk of a resident acquiring an infection as a result of my failure to comply with the hand hygiene guidelines is not very high |
| I do not apply hand hygiene at the sink that is also used by residents |
| Hand hygiene is not a priority at our ward |
| There is a shortest of staff in my ward |
| My colleagues strictly adhere to the hand hygiene guidelines |
| My manager holds team members accountable for hand hygiene performance |
| There is little evidence that hand hygiene prevents infections in residents |
| At my ward, alcohol-based hand rub is in the immediate vicinity |
| My manager supports me in adhering to the hand hygiene guidelines to the best of my ability |
| At our ward, we often have to apply hand hygiene |
| My colleagues think that the hand hygiene prescriptions do not always need to be followed |
| It happens regularly that I have to interrupt a nursing procedure (e.g., if I have forgotten materials) |
| Not performing hand hygiene could have (severe) implications for the patient |
| At my ward, water, soap and towels are in the immediate vicinity |
| At my ward, hand hygiene is only addressed during visits by the Health Inspectorate |
| Hand hygiene during procedures with low risk of contamination is of less importance |
| My manager thinks I should always adhere to the hand hygiene guidelines |
| I perceive the personal hygiene of the residents on my ward as sufficient |
| Not performing hand hygiene could have (severe) implications for me |
| In my ward the access to the sink is hindered by a variety of materials and objects, which makes it difficult to apply hand hygiene |
| Infection prevention is an important topic within my ward |
| There are often busy shifts at my ward |
| At my ward, colleagues support each other in adhering to the hand hygiene guidelines |
| My manager addresses barriers to enable hand hygiene as recommended |
| At my ward, the hand hygiene guidelines is easy to refer to |
| It happens frequently that supplies of soap and towels are not replenished |
| My manager regularly pays attention to adhering the hand hygiene guidelines |
| Correct application of hand hygiene hampers a good relationship with a resident |
| I know exactly how to perform hand hygiene |
| The residents on my ward think I should always follow the hand hygiene guidelines |
| I know the content of the hand hygiene guidelines |
| I know exactly when to perform hand hygiene |
| When I am wearing gloves, I don’t have to perform hand hygiene |
| Other patient safety issues are more important than hand hygiene |
| It happens frequently that supplies of hand alcohol ( bottles/dispensers) are not replenished |
| Sometimes, I don't consider or forget to apply hand hygiene |
| If I notice a colleague not complying to the hand hygiene guidelines, I will call him/her on it |
| During my work, I carry a pocket bottle of hand alcohol |
| The hand hygiene guidelines is evidence-based |
| Our team is informed about our hand hygiene performance |
| The hand hygiene guidelines is not clear |
| At my ward we regularly pay attention to the correct application of hand hygiene |
| Applying hand hygiene is harmful for my skin |
| Applying hand hygiene takes me little time |
| My ward manager provides resources to enable hand hygiene as recommended |
| The hand hygiene guidelines contains too much information |
